# Glycosuria and Renal Outcomes in Patients with Nondiabetic Advanced Chronic Kidney Disease

**DOI:** 10.1038/srep39372

**Published:** 2016-12-23

**Authors:** Chi-Chih Hung, Hugo You-Hsien Lin, Jia-Jung Lee, Lee Moay Lim, Yi-Wen Chiu, Heng-Pin Chiang, Shang-Jyh Hwang, Hung-Chun Chen

**Affiliations:** 1 Division of Nephrology, Department of Internal Medicine, Kaohsiung Medical University Hospital, Kaohsiung Medical University, Kaohsiung, Taiwan; 2 Department of Internal Medicine, Kaohsiung Municipal Ta-Tung Hospital, Kaohsiung Medical University, Kaohsiung, Taiwan; 3 Faculty of Renal Care, College of Medicine, Kaohsiung Medical University, Kaohsiung, Taiwan; 4 Department of Healthcare Administration and Medical Informatics, Kaohsiung Medical University, Kaohsiung, Taiwan; 5 Division of Nephrology, Department of Internal Medicine, Jiannren Hospital, Kaohsiung, Taiwan

## Abstract

Sodium glucose cotransporter 2 inhibitors have shown a potential for renoprotection beyond blood glucose lowering. Glycosuria in nondiabetic patients with chronic kidney disease (CKD) is sometimes noted. Whether glycosuria in CKD implies a channelopathy or proximal tubulopathy is not known. The consequence of glycosuria in CKD is also not studied. We performed a cross-sectional study for the association between glycosuria and urine electrolyte excretion in 208 nondiabetic patients. Fractional excretion (FE) of glucose >4% was 3.4%, 6.3% and 62.5% in CKD stage 3, 4 and 5, respectively. These patients with glycosuria had higher FE sodium, FE potassium, FE uric acid, UPCR, and urine NGAL-creatinine ratio. We conducted a longitudinal study for the consequence of glycosuria, defined by dipstick, in 769 nondiabetic patients with stage 4–5 CKD. Glycosuria was associated with a decreased risk for end-stage renal disease (adjusted hazard ratio: 0.77; CI = 0.62–0.97; *p = 0.024*) and for rapid renal function decline (adjusted odds ratio: 0.63; CI = 0.43–0.95; *p = 0.032*); but glycosuria was not associated with all-cause mortality or cardiovascular events. The results were consistent in the propensity-score matched cohort. Glycosuria is associated with increased fractional excretion of electrolytes and is related to favorable renal outcomes in nondiabetic patients with stage 5 CKD.

Glycosuria is defined as the presence of detectable glucose in the urine. Glucose is almost exclusively reabsorbed by the proximal tubule, through the sodium glucose cotransporter (SGLT) on the apical membrane and the facilitative glucose transporters (GLUT) on the basolateral membrane. The SGLT2 inhibitor is a new category of oral hypoglycemic agent for treating diabetes, which causes substantial glycosuria. Recently, SGLT2 inhibition has shown potential for renoprotection in addition to blood glucose lowering in those with diabetic chronic kidney disease (CKD)^1^,^2^,^3^.

Glycosuria in nondiabetic patients having a euglycemic status is a sign of an impaired renal proximal tubular reabsorption of glucose. In the limited case reports and animal models of familial renal glycosuria, glycosuria has not caused renal dysfunction^4^,^5^,^6^. No significant renal impairment has been reported in the short-term reports on SGLT2 inhibitor treatment^2^,^7^,^8^. However, the long-term consequences of glycosuria are not well understood. Glycosuria is sometimes observed in patients with advanced CKD even in the absence of diabetes. The relevance of glycosuria in these patients is uninvestigated, and an impairment of proximal tubular reabsorption is hypothesized.

By contrast, the proximal tubular reabsorption of filtered glomerular proteins could cause renal tubular injury. The reabsorbed proteins, which accumulate within proximal tubular epithelial cells (PTECs), activate cytokines and a series of pathological changes, thus inducing a decline in renal function^9^,^10^. Impaired renal proximal tubular reabsorption of proteins from glomerular filtrates might prevent the proximal tubule from intracellularly accumulating the filtered proteins and subsequent renal tubular injury^11^,^12^.

We hypothesized that, first, glycoscuria is a sign for proximal tubulopathy and, second, glycosuria could be associated with more favorable renal outcomes in patients with nondiabetic CKD and proteinuria. We tested these 2 hypotheses in an observational cohort study.

## Results

### Renal Handling of Electrolytes and Glucose in Nondiabetic Patients Stratified by CKD Stages and Factors Associated with Fractional Excretion of Glucose in the Cross-Sectional Study

In the cross-sectional study of 208 patients, 145 (69.7%) had nondiabetic CKD (Table 1). Fractional excretion (FE) of glucose, FE sodium, FE potassium, and FE uric acid were higher in the patients with stage 4 or 5 CKD than in those without CKD or with stage 1–3 CKD. The percentage of glycosuria measured using a dipstick was 56.3% and that measured using FE glucose of >4% was 62.5% in patients with stage 5 CKD. (Table 1) Glycosuria measured using a dipstick and equivalent FE glucose were shown in Supplementary Table 1. Other characteristics of these patients were shown in Supplementary Table 2.

**Figure 1:**
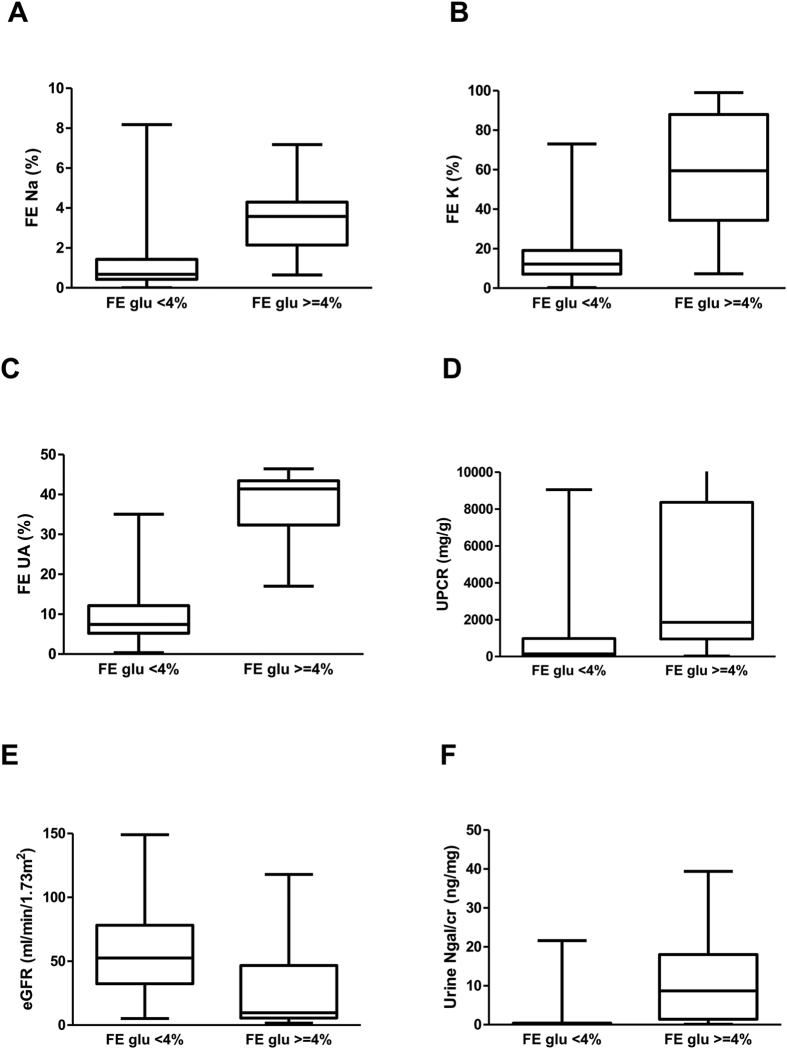
The median values and interquartile ranges of fractional excretion of sodium (FE Na), postassium (FE K), uric acid (FE UA), urine protein-creatinine ratio (UPCR), estimated glomerular filtration rate (eGFR) and urine neutrophil gelatinase-associated lipocalin – creatinine ratio (Ngal/Cr) in patients with FE glucose <4% and FE glucose ≥4%. All *P* values < 0.05 for Mann-Whitney U test between two groups through A to F.

The patients with FE glucose ≥4% had higher FE sodium, FE potassium, FE uric acid, urine protein-creatinine ratio (UPCR), and urine neutrophil gelatinase-associated lipocalin (NGAL)-creatinine ratio than those with FE glucose <4%, as displayed in Fig. 1. All P values < 0.05 for Mann-Whitney U test between two groups through Fig. 1 A to F. In logistic regression analysis, FE glucose ≥4% was associated with FE sodium and eGFR (Table 2).

### Characteristics of Patients with Nondiabetic Stage 4–5 CKD Stratified by Glycosuria and Factors Associated with Glycosuria in the Longitudinal Study

In the longitudinal study of 769 patients with nondiabetic stage 4–5 CKD, 279 (36.3%) had glycosuria measured using a dipstick (Table 3). The mean age of these patients was 61.3 years, and the median eGFR was 12.1 mL/min per 1.73 m^2^. The glycosuria group had fewer men, lower percentage of hyperuricemia or cardiovascular disease, and lower percentage of patients who took RAS blockers, other antihypertensives, statins, or aspirin. The glycosuria group had lower eGFR, serum hemoglobin, body mass index (BMI), total cholesterol, triglyceride, potassium, calcium, bicarbonate, and uric acid level than did the nonglycosuria group. Moreover, the glycosuria group had higher UPCR and serum phosphorus. The glycosuria and nonglycosuria groups did not differ in age, percentage of baseline hypertension, mean BP, serum albumin, and sodium level. After propensity score matching, the glycosuria group had lower serum uric acid, triglyceride, calcium and higher glucose level. All other characteristics were not different between glycosuria group and nonglycosuria group (Supplementary Table 3).

### Glycosuria, End-Stage Renal Disease (ESRD), and Rapid Renal Function Decline

Over a median follow-up period of 3.1 years, 385 patients progressed to long-term hemodialysis, peritoneal dialysis, or renal transplantation (Table 4). The crude event rate of ESRD per 100 patient-years was 18.13 and 11.00 in the glycosuria and nonglycosuria groups, respectively. In Cox regression analysis, the glycosuria group had a higher unadjusted risk of ESRD; the hazard ratio (HR) was 1.78 (95% confidence interval [CI]: 1.45–2.18). However, after adjustment for age, sex, eGFR, UPCR and causes of CKD in Model 1, the glycosuria group had a lower risk of ESRD than did the nonglycosuria group. Furthermore, in the fully adjusted model, the glycosuria group had a lower risk of ESRD; the HR was 0.77 (0.95% CI: 0.62–0.97). The multivariate logistic regression analysis of rapid renal function decline by glycosuria is provided in Table 4. Rapid renal function decline was defined as an eGFR slope of less than −5 mL/min/1.73 m^2^/y according to the KDIGO guidelines^13^. In the fully adjusted model, the odds ratio of rapid renal function decline in the glycosuria group was 0.63 (95% CI: 0.43–0.95) compared with that in the nonglycosuria group. In the propensity score matched cohort, the fully adjusted HR of ESRD in glycosuria group was 0.78 (95% CI: 0.62–0.98) compared with that in the nonglycosuria group. The OR of rapid renal function decline in glycosuria group was 0.64 (95% CI: 0.41–0.99) in the fully adjusted model (Supplementary Table 4).

### Glycosuria, All-Cause Mortality, and Cardiovascular Events

Overall, 138 patients died (Table 4). The crude event rate per 100 patient-years was 3.70 and 4.73 in the glycosuria and nonglycosuria groups, respectively. In fully adjusted Cox regression, the HR of all-cause mortality in the glycosuria group was 0.92 (95% CI: 0.62–1.37) compared with that in the nonglycosuria group. Furthermore, 107 patients experienced cardiovascular (CV) events. The crude event rate per 100 patient-years was 2.89 in the glycosuria group and 3.65 in the nonglycosuria group. In fully adjusted Cox regression, the HR of CV events in the glycosuria group was 0.88 (95% CI: 0.56–1.37) compared with that in the nonglycosuria group. In the propensity score matched cohort, the fully adjusted HR of all-cause mortality in the glycosuria group was 0.89 (95% CI: 0.58–1.36) compared with that in the nonglycosuria group. The fully adjusted HR of CV events in the glycosuria group was 0.88 (95% CI: 0.55–1.41) compared with that in the nonglycosuria group (Supplementary Table 4).

## Discussion

In this study, we observed that glycosuria is common in patients with nondiabetic stage 4–5 CKD. We also revealed these patients with glycosuria had higher FE sodium, FE potassium, FE uric acid, UPCR, and urine NGAL-creatinine ratio. We demonstrated, for the first time, that glycosuria was associated with a lower risk of ESRD (0.77-fold) and rapid renal function decline (0.63-fold) in patients with nondiabetic stage 5 CKD. The results were consistent in the propensity-score matched cohort.

Glycosuria commonly occurs in patients with diabetes when the amount of the filtered glucose exceeds the capacity of renal tubular reabsorption. The role of glycosuria as a screening tool is very limited because of its low sensitivity^14^ and the high individual variability of the renal threshold of glucose excretion^15^. The effects of glycosuria on patients with poorly controlled diabetes have been previously proposed^16^,^17^. Glycosuria induces osmotic diuresis and is thus concerning. Recently, the association of glycosuria and clinical outcomes has again generated interest because of the use of SGLT2 inhibitors. Short-term trials of SGLT2 inhibitors have reported that, in addition to reducing blood glucose, SGLT2 inhibitors have other potential benefits, including blood pressure reduction, diuretic effects, body weight reduction, and uric acid reduction^1^,^2^,^7^. One hypothesis has been proposed that, SGLT2 inhibition could restore the tubuloglomerular feedback and reduce hyperfiltration in diabetic nephropathy^2^.

Glycosuria is rarely observed in the general population and may be found in patients with nondiabetic CKD. Our study was the first to investigate glycosuria in this population, and it showed that glycosuria becomes relatively frequent with renal function decline. However, glycosuria in patients with nondiabetic CKD in our study might be associated with proximal tubulopathy rather than merely SGLT channelopathy. First, the median FE glucose was 10% in our patients with glycosuria, which was lower than that in the patients to whom full-dose SGLT2 inhibitors were administered (approximately 30% in clinical trials)^18^,^19^. Second, simultaneously, the median FE Na was 3.4% in our patients with glycosuria, which was higher than that in the patients to whom full-dose SGLT2 inhibitors were administered (<1% in clinical trials)^19^,^20^. The median FE UA was also higher in our patients with glycosuria than in the patients to whom full-dose SGLT2 inhibitors were administered^21^. These data suggested that the urine excretion of electrolytes in our study exceeds the effect of SGLT2 inhibition. The mechanism underlying the proximal tubulopathy in those with advanced CKD remains unclear. We hypothesized from the remnant nephron theory that in patients with proximal tubulopathy, the reabsorption in proximal tubules could not match the hyperfiltration from the glomeruli.

The effects of glycosuria on patients with nondiabetic CKD are largely unknown. Our study was the first to examine the factors associated with in nondiabetic patients. We also observed a lower blood pressure, serum uric acid, and BMI in patients with glycosuria, similar to the findings in SGLT2 inhibitor trials. Our study was also the first to examine the clinical outcomes of patients with glycosuria. Our data suggested that, in the patients with nondiabetic stage 5 CKD, glycosuria associates with lower risk of ESRD and lower risk of rapid renal function decline. Current trials of SGLT2 inhibitors exclude patients with advanced CKD. Large-scale studies are warranted to study the cause and effect of glycosuria in patients with nondiabetic CKD.

However, because only low-grade glycosuria occurs in patients with nondiabetic CKD in our study, and the glycosuria is related to increased fractional excretion of electrolytes in these patients, we propose another hypothesis. That is, proximal tubulopathy might play a role on the favorable renal outcomes in these patients. In patients with CKD and proteinuria, filtered proteins are mainly reabsorbed and accumulated within PTECs, causing tubulointerstitial inflammation and fibrogenesis by activating immunoregulatory cytokines and vasoactive genes, such as endothelin-1 and regulated upon activation normal T-cell expressed and secreted^9^,^10^. The protein traffic induces PTECs to acquire an inflammatory phenotype both *in vitro*^22^,^23^ and *in vivo*^24^,^25^. Another study reported that albumin-induced reactive oxygen species generation, nuclear factor kappa beta activation, and interleukin-8 secretion are endocytosis dependent^12^. In animal studies, limiting the protein traffic has been shown to prevent renal disease progression^11^,^26^,^27^. Thus, proximal tubulopathy might associate with favorable renal outcomes in patients with CKD.

Regarding the association between glycosuria and CV events, our results revealed no difference between the glycosuria and nonglycosuria groups (Table 4). The EMPA-REG OUTCOME study reported that empagliflozin intervention was associated with decreased CV events in patients with type 2 diabetes mellitus having a high CV risk^22^. However, the patients in our study were nondiabetic and had advanced CKD with low-grade glycosuria; therefore, the effect might have differed. Regarding the association between glycosuria and all-cause mortality, our study revealed no difference between the glycosuria and nonglycosuria groups (Table 4). No previous study has investigated this association.

This study had several limitations. First, this was an observational study, and causal relationships thus could not be delineated. Second, dipstick urinalysis cannot always reveal an accurate concentration of glucose in the urine because of the influences of certain substances, such as ascorbic acid and strong oxidizing agents^23^, despite this, the sensitivity, specificity, and positive and negative predictive values of the dipstick test for detecting glucose were 100%, 98.5%, 87%, and 100%, respectively, in a previous study^24^. Third, we could not determine whether the favorable renal outcomes were specifically associated with glycosuria, proximal tubulopathy, or both. Fourth, glycosuria did not exhibit a dose-dependent effect because of the limited number of events. Fifth, CV events might be underestimated. Sixth, although we did the analysis in a propensity-score matched cohort, the confounding could not be completely eliminated.

In conclusion, we found the association between glycosuria and increased fractional excretion of electrolytes in nondiabetic patients with advanced CKD. We also demonstrated that glycosuria is associated with favorable renal outcomes. However, the cause and mechanism of glycosuria remain unclear. Large scale studies are necessary to clarify this phenomenon.

## Methods

### Patients and Measurements

From November 11, 2002 to May 31, 2009, 3749 patients with stage 1–5 CKD were included from the Integrated CKD Care Program in Kaohsiung for Delaying Dialysis. This observational study was conducted at 2 affiliated hospitals of Kaohsiung Medical University in Southern Taiwan. All the patients were followed until May 31, 2010 or death, as previously reported^28^. To investigate the renal handling of electrolyte and glucose, we conducted a cross-sectional study which was designed to include 60 nondiabetic patients without CKD and 30 patients in each CKD groups (CKD stage 1–2, CKD 3a, CKD 3b, CKD 4 and CKD 5) from the nephrology outpatient department. Finally, we included 63 nondiabetic patients without CKD and 145 nondiabetic patients with CKD. Furthermore, to investigate the association between glycosuria and clinical outcomes, we selected 769 patients with nondiabetic stage 4–5 CKD and a UPCR of ≥500 mg/g in the longitudinal study. DM was diagnosed on the basis of the treatment administered or a glycated hemoglobin level of ≥6.5% at the time of enrollment. The study protocol was approved by the Institutional Review Board of Kaohsiung Medical University Hospital (KMUH-IRB-990198), and all the patients provided written informed consent for study participation. The methods were performed in accordance with relevant guidelines and regulations.

The baseline characteristics of all the patients included demographic data, comorbidities, medication history, lifestyle factors, physical examination findings, and laboratory data. Glycosuria was defined as a urine glucose level of ≥1+ at 2 or more times in 3 consecutive urine analyses at the outpatient department with at least one week interval within 3 months. The samples exhibiting >5 white blood cells per high power field (hpf) or >5 red blood cells per hpf in urinalysis were excluded. Furthermore, urine glucose and electrolytes and the corresponding serum data were collected on the same day for measuring FE. Patient demographic data were recorded at the first visit, and their medical histories were recorded according to a chart review. Hypertension was defined on the basis of clinical diagnoses and the prescribed medications. CV diseases were defined according to the clinical diagnoses of heart failure, acute or chronic ischemic heart disease, or cerebrovascular disease. Moreover, laboratory data were obtained at the first visit.

### Clinical Outcomes

Four clinical outcomes, namely ESRD, rapid renal function decline, CV events, and all-cause mortality, were assessed. ESRD was defined as the initiation of maintenance hemodialysis, peritoneal dialysis, or renal transplantation. Moreover, ESRD was ascertained according to a chart and catastrophic card review. A rapid renal function decline was defined as an eGFR slope of less than −5 mL/min/1.73 m^2^/y on the basis of Kidney Disease Improving Global Outcomes (KDIGO) guidelines. The eGFR was defined using the simplified Modification of Diet in Renal Disease Study equation: eGFR (mL/min/1.73 m^2^) = (186) × (serum creatinine − 1.154) × (age − 0.203) × (C), where C is 0.742 for women, 1.212 for African American patients, and 1 for other patients. In addition, CV events were ascertained by reviewing charts to identify hospitalization for acute coronary syndrome, acute cerebrovascular disease, congestive heart failure, and peripheral arterial occlusion disease and death resulting from any of the aforementioned causes. The survival status and cause of death were determined by reviewing death certificates, patient charts, and the National Death Index.

### Statistical Analysis

The summarized statistical results of the baseline characteristics of all the patients and stratification by glycosuria status were expressed as percentages for categorical data, mean ± standard deviation for continuous variables with an approximately normal distribution, and median and interquartile ranges for continuous variables with a skewed distribution. Moreover, linear regression analysis was performed to study the association between FE glucose and other parameters. Cox proportional hazards analysis was used to assess the association between glycosuria and the clinical outcomes. Multivariate logistic regression analysis was used to evaluate the association between glycosuria and rapid renal function decline. The covariates were selected according to our previous studies^29^. The continuous variables with skewed distributions were log-transformed to reduce the skewness. The fully adjusted model was adjusted for age; sex; causes of CKD, eGFR, log-transformed UPCR, cholesterol, and C-reactive protein; baseline hypertension and CV disease; mean blood pressure; hemoglobin; albumin; BMI; and phosphorus. *P* < 0.05 was considered statistically significant. The models for all-cause mortality were censored only at death or the end of follow-up. Moreover, the models for CV events were censored at the development of these events, death, or the end of follow-up. The models for ESRD were censored at the commencement of renal replacement therapy, death, or the end of follow-up.

The propensity score is the conditional probability of receiving an exposure given a set of measured covariates. We estimated propensity scores for glycosuria for each of the 769 patients using a non-parsimonious multivariable logistic regression model including all parameters shown in Table 1. The model was well-calibrated (Hosmer–Lemeshow test: P = 0.167) with reasonable discrimination (c statistic = 0.68). We matched patients in glycosuria group with patients in non-glycosuria group who had similar propensity scores to five, four, three, two and one decimal places in five repeated steps. In the first step, we multiplied the raw propensity scores by 100 000, then rounded it to the nearest value. This was repeated, multiplying by 10000, 1000, 100, and 10. Statistical analysis was performed using R 3.3.0 software (R Foundation for Statistical Computing, Vienna, Austria) and Statistical Package for Social Sciences, Version 21.0, for Windows (SPSS Inc., Chicago, IL, USA).

## Additional Information

**How to cite this article**: Hung, C.-C. *et al*. Glycosuria and Renal Outcomes in Patients with Nondiabetic Advanced Chronic Kidney Disease. *Sci. Rep.* **6**, 39372; doi: 10.1038/srep39372 (2016).

**Publisher's note:** Springer Nature remains neutral with regard to jurisdictional claims in published maps and institutional affiliations.

## Supplementary Material

Supplementary Information

## Figures and Tables

**Table 1:** Electrolytes and glucose renal handling and characteristics of nondiabetic patients by CKD stages in the cross-sectional study.

Variables	All patients	Non-CKD	CKD				*P* value
			Stage 1 & 2	Stage 3	Stage 4	Stage 5	
Patients, n	208	63	23	58	32	32	
**Demographic data**							
Age, mean (SD), y	57 (15.2)	53.5 (13.4)	45.8 (18.8)	60.1 (13.5)	62.9 (14.8)	60.4 (14.2)	<0.001
Male, n (%)	74 (35.6)	6 (26.1)	28 (44.4)	18 (31)	12 (37.5)	10 (31.3)	0.007
SBP, mean (SD), mmHg	129.8 (17.1)	126.7 (15)	123.4 (14.5)	127.8 (17.7)	129.9 (14.6)	144.0 (17.2)	<0.001
eGFR, mean (SD), ml/min/1.73 m^2^ a	52.5 (33.8)	83.5 (17.3)	92.4 (30)	43.8 (8.4)	21.6 (3.7)	9.6 (9.2)	<0.001
Urine PCR, median (IQR), mg/g	201 (87–1323)	80 (51–114)	772 (641–1278)	127 (84–617)	1043 (283–1496)	1814 (1400–4745)	<0.001
**Renal tubular function**							
FE glucose, median (IQR), %	0.1 (0.1–0.4)	0.1 (0.1–0.1)	0.1 (0.1–0.2)	0.1 (0.1–0.2)	0.3 (0.2–0.7)	6.8 (1.8–25.2)	<0.001
FE Na, median (IQR), %	0.7 (0.4–1.9)	0.5 (0.3–0.6)	0.4 (0.2–1.1)	0.7 (0.5–1.2)	1.9 (1.7–2.1)	3.6 (2.9–6.6)	<0.001
FE K, median (IQR), %	13.1 (8.4–22.9)	8.4 (6.3–12.0)	10.0 (6.3–28.4)	12.8 (9.9–15.8)	19.4 (13.5–26.6)	44.2 (28.1–93.2)	<0.001
FE UA, median (IQR), %	7.8 (5.2–14.9)	5.7 (3.8–8.5)	7.4 (3.5–11.7)	7.3 (4.5–9.4)	15.0 (6.2–19.0)	36.2 (21.6–45.6)	<0.001
Glycosuria by dipstick, n (%)	22 (10.6)	0	0	2 (3.4)	2 (6.3)	18 (56.3)	<0.001
FE glucose >1%, n (%)	36 (17.3)	1 (1.6)	2 (8.7)	2 (3.4)	4 (12.5)	27 (84.4)	<0.001
FE glucose >4%, n (%)	25 (12.0)	1 (1.6)	0	2 (3.4)	2 (6.3)	20 (62.5)	<0.001
Urine NGAL-to-creatinine (ng/mg)	0.01 (0.00–0.01)	0.03 (0.01–0.05)	0.11 (0.52–0.23)	0.24 (0.14–0.50)	1.16 (0.17–2.43)	13.84 (3.64–30.94)	<0.001
**Biochemistry and electrolytes**							
Blood glucose, mean (SD), mg/dL	101.1 (18)	100.1 (15.3)	97.2 (18.2)	101.5 (13.0)	104.4 (26.7)	102.1 (20.0)	<0.001
Sodium, mean (SD), mEq/L	139.2 (4.1)	139.2 (4.1)	139.3 (4.1)	139.8 (4.0)	138.1 (4.3)	139.2 (4.3)	0.002
Potassium, mean (SD), mEq/L	4.1 (0.7)	3.9 (0.3)	3.8 (0.8)	4.1 (0.5)	4.4 (1.0)	4.7 (0.7)	<0.001
Calcium, mean (SD), mg/dL	9.0 (0.8)	9.2 (0.9)	9.0 (0.6)	9.2 (0.8)	8.9 (0.6)	8.4 (0.5)	<0.001
Phosphorus, mean (SD), mg/dL	4.1 (1.5)	3.6 (1.9)	3.9 (0.8)	4.0 (1.4)	4.3 (1.5)	4.9 (1.1)	<0.001
Uric acid, mean (SD), mg/dL	7.2 (1.8)	6.4 (1.5)	7.1 (1.6)	7.6 (1.9)	7.9 (2.0)	7.8 (1.5)	<0.001

Abbreviations: CKD, chronic kidney disease; eGFR, estimated glomerular filtration rate; FE glucose, fraction excretion of glucose; FE K, fraction excretion of potassium; FE Na, fraction excretion of sodium; FE UA, fraction excretion of uric acid; Hba1c, glycosylated hemoglobin; IQR, interquartile range; NGAL, neutrophil gelatinase-associated lipocalin; SBP, systolic blood pressure; SD, standard deviation; UPCR, urine protein-creatinine ratio.

P < 0.05 indicates a significant difference among groups by ANOVA.

^a^Obtained by using the Modification of Diet in Renal Disease study equation.

**Table 2:** Logistic regression for FE glucose ≧4% in non-diabetic CKD patients in the cross-sectional study.

Variables	Odds ratio	95% CI	*p*-value
Age	1.005	0.957 to 1.056	0.834
Gender (male)	0.646	0.156 to 2.678	0.547
eGFR (ml/min/1.73 m^2^)	0.941	0.887 to 0.998	0.042
Log UPCR	1.336	0.308 to 5.794	0.699
Cardiovascular disease	0.958	0.782 to 1.581	0.678
SBP, mmHg	0.934	0.605 to 1.441	0.756
Body weight, kg	0.963	0.919 to 1.009	0.115
Hemoglobin, g/dL	0.875	0.531 to 1.442	0.600
Albumin, g/dL	1.179	0.172 to 8.090	0.867
FE sodium, %	1.039	1.002 to 1.084	0.026
Phosphorus, g/dL	1.109	0.657 to 1.873	0.698
Bicarbonate, mEq/L	1.031	0.849 to 1.251	0.758

Abbreviations: eGFR, estimated glomerular filtration rate; FE, fraction excretion; Log, logarithm; SBP, systolic blood pressure; UPCR, Urine protein-creatinine ratio.

**Table 3:** Characteristics of patients with nondiabetic stage 4–5 CKD by glycosuria in the longitudinal cohort study.

Variable	All	Non-glycosuria	Glycosuria	*p*-value
Patients, n	769	490	279	
**Demographics and Medical History**				
Age, mean (SD), y	61.3 (15.2)	61.6 (15.8)	60.6 (14.2)	0.680
Male, n (%)	351 (45.6)	242 (49.4)	109 (39.1)	0.006
BMI, mean (SD), Kg/m^2^	24.0 (4.2)	24.3 (4.2)	23.5 (4.2)	0.011
MBP, mean (SD), mmHg	101.1 (14.3)	101.6 (14.7)	100.4 (13.6)	0.267
Smoker, n (%)	56 (7.3)	40 (8.2)	16 (5.7)	0.213
Hypertension, n (%)	482 (62.7)	304 (62.0)	178 (63.8)	0.628
Hyperuricemia, n (%)	137 (17.8)	109 (22.2)	28 (10.0)	<0.001
Cardiovascular disease, n (%)	145 (18.9)	103 (21.0)	42 (15.1)	0.042
Causes of chronic kidney diseases^a^				0.095
Primary glomerular disease	459 (59.7)	278 (56.7)	181 (64.9)	
Tubulointerstitial nephropathy	157 (20.4)	106 (21.6)	51 (18.3)	
Hypertensive nephrosclerosis	111 (14.4)	80 (16.3)	31 (11.1)	
Others	42 (5.5)	26 (5.3)	16 (5.7)	
**Medication**				
RAS blockers, n (%)	289 (37.6)	222 (45.3)	67 (24.0)	<0.001
Other antihypertensives, n (%)	269 (35.0)	197 (40.2)	72 (25.8)	<0.001
Statins, n (%)	110 (14.3)	81 (16.5)	29 (10.4)	0.019
Diuretics, n (%)	124 (16.1)	81 (16.5)	43 (15.4)	0.685
Beta-blockers, n (%)	128 (16.6)	92 (18.8)	36 (12.9)	0.036
Aspirin, n (%)	45 (8.4)	24 (9.0)	21 (7.9%)	0.027
**Renal Function Status**				
eGFR, median (IQR), ml/min/1.73 m^2^ b	12.1 (7.3–19.0)	14.8 (9.3–21.3)	8.4 (5.8–12.6)	<0.001
Urine PCR, median (IQR), mg/g	1406 (950–2308)	1303 (890–2207)	1579 (1050–2461)	<0.001
CKD stage				<0.001
Stage 4	288 (37.5)	241 (49.2)	47 (16.8)	
Stage 5	481 (62.5)	249 (50.8)	232 (83.2)	
**Laboratory Data**				
Hemoglobin, mean (SD), g/dL	9.7 (1.9)	10.1 (2.0)	9.0 (1.6)	<0.001
Albumin, mean (SD), g/dL	3.9 (0.5)	3.9 (0.5)	3.9 (0.5)	0.552
Blood glucose, mean (SD), mg/dl	98.2 (15.0)	97.2 (14.2)	99.6 (16.4)	0.037
Total cholesterol, median (IQR), mg/dL	187 (158–215)	190 (160–222)	184 (153–210)	0.024
Triglyceride, median (IQR), mg/dL	115 (81–163)	123 (85–170)	101 (75–149)	<0.001
C-reactive protein, median (IQR), mg/L	1.2 (0.5–5.1)	1.1 (0.4–5.5)	1.4 (0.5–5.0)	0.098
Hba1c, %	5.4 (0.6)	5.4 (0.6)	5.3 (0.6)	0.003
Sodium, mean (SD), mEq/L	138.2 (3.4)	138.3 (3.5)	138.1 (3.1)	0.425
Potassium, mean (SD), mEq/L	4.4 (0.6)	4.5 (0.6)	4.4 (0.6)	0.008
Phosphorus, mean (SD), mg/dL	4.9 (1.4)	4.8 (1.4)	5.2 (1.4)	<0.001
Calcium, mean (SD), mg/dL	8.9 (0.8)	9.0 (0.8)	8.7 (0.9)	<0.001
Bicarbonate, mean (SD), mEq/L	19.1 (4.3)	20.0 (4.2)	17.4 (3.9)	<0.001
Uric acid, mean (SD), mg/dL	8.0 (2.0)	8.5 (2.0)	7.1 (1.7)	<0.001

Abbreviations: BMI, body mass index; CKD, chronic kidney disease; CRP, c-reactive protein; eGFR, estimated glomerular filtration rate; Hba1c, glycosylated hemoglobin; IQR, interquartile range; MBP, mean blood pressure; RAS, renin-angiotensin system; SD, standard deviation; UPCR: urine protein-creatinine ratio.

^a^The distribution of the causes of chronic kidney diseases is similar to the national epidemiologic data in Taiwan ( http://www.tsn.org.tw/UI/K/K008.aspx).

^b^Obtained by using the Modification of Diet in Renal Disease study equation.

**Table 4:** Risk of clinical outcomes by glycosuria status in patients with nondiabetic stage 4–5 CKD^a^.

	Events (%)	Crude Event Rate (/100 patient-years)	Risk^d^		
			Unadjusted	Model 1	Fully adjusted model
**ESRD**^b^					
Non-glycosuria	214 (43.7)	11.00	1 (reference)	1 (reference)	1 (reference)
Glycosuria	171 (61.3)	18.13	1.78 (1.45–2.18)	0.70 (0.56–0.87)	0.77 (0.62–0.97)
**Rapid Renal Function Decline**^c^					
Non-glycosuria	134 (27.3)	6.89	1 (reference)	1 (reference)	1 (reference)
Glycosuria	50 (17.9)	4.02	0.59 (0.41–0.85)	0.61 (0.40–0.91)	0.63 (0.43–0.95)
**Cardiovascular events**					
Non-glycosuria	71 (14.5)	3.65	1 (reference)	1 (reference)	1 (reference)
Glycosuria	36 (12.9)	2.89	0.96 (0.64–1.43)	0.79 (0.52–1.22)	0.88 (0.56–1.37)
**All-cause Mortality**					
Non-glycosuria	92 (18.8)	4.73	1 (reference)	1 (reference)	1 (reference)
Glycosuria	46 (16.5)	3.70	0.87 (0.61–1.24)	0.94 (0.64–1.38)	0.92 (0.62–1.37)

Abbreviations: BMI, Body mass index; CKD, Chronic kidney disease; CI, Confidence interval; HR, Hazard ratio; UPCR, Urine protein-creatinine ratio.

^a^Model 1 adjusts for age, gender, eGFR, log-transformed UPCR, and causes of CKD; fully adjusted model adjusts for covariates in model 1 plus cardiovascular disease history, mean blood pressure, hemoglobin, albumin, log-transformed CRP, BMI, log-transformed cholesterol, phosphorus, uric acid, potassium and bicarbonate.

^b^ESRD includes long-term hemodialysis, peritoneal dialysis, and renal transplantation.

^c^defined as eGFR slope <−5 ml/min/1.73 m^2^/year based on Kidney Disease Improving Global Outcomes (KDIGO) guideline.

^d^Hazard ratio of ESRD, cardiovascular events, and all-cause mortality; odds ratio of rapid renal function decline.
