# Supplementary material for: Glycosuria and Renal Outcomes in Patients with Nondiabetic Advanced Chronic Kidney Disease
Source: Sci Rep. 2016 Dec 23;6:39372. doi: 10.1038/srep39372 (PMC5180243; doi:10.1038/srep39372)
Supplement: Supplementary Information [file srep39372-s1.doc]

**Glycosuria and Renal Outcomes in Patients with Nondiabetic Advanced Chronic Kidney Disease**

Chi-Chih Hung, MD^1^, Hugo You-Hsien Lin^1,2^, MD, Jia-Jung Lee, MD^1^, Lee Moay Lim, MD^1^, Yi-Wen Chiu, MD^1,3^, Heng-Pin Chiang, MD^*4,5^, Shang-Jyh Hwang, MD^1,3^, Hung-Chun Chen, MD, PhD^1,3^

^1^Division of Nephrology, Department of Internal Medicine, Kaohsiung Medical University Hospital, Kaohsiung Medical University, Kaohsiung, Taiwan

^2^Department of Internal Medicine, Kaohsiung Municipal Ta-Tung Hospital, Kaohsiung Medical University, Kaohsiung, Taiwan

^3^Faculty of Renal Care, College of Medicine, Kaohsiung Medical University, Kaohsiung, Taiwan

^4^Department of Healthcare Administration and Medical Informatics, Kaohsiung Medical University, Kaohsiung, Taiwan

^5^Division of Nephrology, Department of Internal Medicine, Jiannren Hospital, Kaohsiung, Taiwan

*Supplementary Table 1.* The relationship between dipstick glycosuria and fractional excretion of glucose in nondiabetic patients

| **Dipstick glycosuria** |  | **FE glucose (%)** | **Cases of FE glucose > 1%/ Cases of dipstick result** | **Cases of FE glucose > 4%/ Cases of dipstick result** |
| --- | --- | --- | --- | --- |
| - |  | 0.1 (0.1-0.2) | 15/186 | 4/186 |
| **+** |  | 6.9 (6.1-10.4) | 10/10 | 10/10 |
| ++ |  | 12.8 (7.6-25.8) | 6/6 | 6/6 |
| +++ |  | 20.1 (9.8-39.7) | 6/6 | 6/6 |

Abbreviations: FE, fractional excretion

*Supplementary Table 2.* Characteristics of patients without CKD and patients with CKD in the cross-sectional study

| **Variable** |  | | **Patients without CKD** | **Patients with CKD** | ***p*-value** |
| --- | --- | --- | --- | --- | --- |
| Patients, n |  | | 63 | 145 |  |
| **Demographics and Medical History** | | |  |  |  |
| Age, mean (SD), y | |  | 53.5 (13.4) | 58.5 (15.7) | 0.030 |
| Male, n (%) | |  | 28 (44.4) | 46 (31.7) | 0.078 |
| Hypertension, n (%) | |  | 25 (39.7) | 55 (37.9) | 0.348 |
| Hyperuricemia, n (%) | |  | 12 (19.0) | 26 (17.9) | 0.848 |
| Cardiovascular disease, n (%) | |  | 4 (6.35) | 14 (9.66) | 0.184 |
| **Medication** | |  |  |  |  |
| RAS blockers, n (%) | |  | 19 (30.2) | 69 (47.6) | 0.019 |
| Other antihypertensives, n (%) | |  | 95 (35.6) | 90 (33.7) | 0.340 |
| Statins, n (%) | |  | 2 (3.2) | 4 (2.8) | 0.869 |
| Diuretics, n (%) | |  | 2 (3.2) | 6 (4.1) | 0.101 |
| **Laboratory Data** | |  |  |  |  |
| eGFR, mean (SD), ml/min/1.73 m^2^ ^a^ | |  | 78.6 (69.4-99.1) | 34.9 (17.8-60.0) | <0.001 |
| WBC, mean (SD), x1000cells/μl | |  | 6.2 (1.7) | 6.6 (2.4) | 0.100 |
| Hemoglobin, mean (SD), g/dL | |  | 13.8 (1.1) | 11.7 (2.3) | <0.001 |
| Albumin, mean (SD), g/dL | |  | 4.2 (0.3) | 4.1 (0.5) | 0.151 |
| Blood glucose, mean (SD), mg/dl | |  | 100.1 (15.3) | 101.6 (19.0) | 0.587 |
| Total cholesterol, median (IQR), mg/dL | |  | 181 (148-208) | 174 (147-201) | 0.116 |
| Triglyceride, median (IQR), mg/dL | |  | 96 (64-140) | 97 (66-149) | 0.744 |
|  | |  |  |  |  |

Abbreviations: CKD, chronic kidney disease; eGFR, estimated glomerular filtration rate; IQR, interquartile range; RAS, renin-angiotensin system; SD, standard deviation; WBC, white blood cell

^a^ Obtained by using the Modification of Diet in Renal Disease study equation

*Supplementary Table 3.* Characteristics of patients with nondiabetic stage 4-5 CKD by glycosuria after propensity score matching in the longitudinal cohort study

| **Variable** | **All** | | **Non-glycosuria** | **Glycosuria** | ***p*-value** |
| --- | --- | --- | --- | --- | --- |
| Patients, n | 534 | | 267 | 267 |  |
| **Demographics and Medical History** | | |  |  |  |
| Age, mean (SD), y | | 60.9 (15.0) | 61.2 (15.4) | 60.6 (14.5) | 0.635 |
| Male, n (%) | | 227 (42.5) | 119 (44.6) | 108 (40.4) | 0.336 |
| BMI, mean (SD), Kg/m^2^ | | 23.6 (4.0) | 23.7 (3.8) | 23.6 (4.2) | 0.817 |
| MBP, mean (SD), mmHg | | 100.5 (13.7) | 100.6 (13.6) | 100.3 (13.8) | 0.839 |
| Smoker, n (%) | | 32 (6.0) | 15 (5.6) | 17 (6.4) | 0.715 |
| Hypertension, n (%) | | 326 (61.0) | 159 (59.6) | 167 (62.5) | 0.478 |
| Hyperuricemia, n (%) | | 65 (12.2) | 38 (14.2) | 27 (10.1) | 0.047 |
| Cardiovascular disease, n (%) | | 87 (16.3) | 43 (16.1) | 44 (16.5) | 0.907 |
| Etiologies of chronic kidney diseases | |  |  |  | 0.869 |
| Primary glomerular disease | | 333 (62.4) | 165 (61.8) | 168 (62.9) |  |
| Tubulointerstitial nephropathy | | 98 (18.4) | 50 (18.7) | 48 (18.0) |  |
| Hypertensive nephropathy | | 66 (12.7) | 33 (12.4) | 33 (12.4) |  |
| Others | | 37 (6.9) | 19 (7.1) | 18 (6.7) |  |
| **Medication** | |  |  |  |  |
| RAS blockers, n (%) | | 200 (37.5) | 105 (39.3) | 95 (35.6) | 0.108 |
| Other antihypertensives, n (%) | | 185 (34.6) | 95 (35.6) | 90 (33.7) | 0.340 |
| Statins, n (%) | | 73 (13.7) | 43 (16.1) | 30 (11.2) | 0.102 |
| Diuretics, n (%) | | 72 (13.5) | 35 (13.1) | 43 (16.1) | 0.361 |
| Beta-blockers, n (%) | | 80 (15) | 43 (16.1) | 37 (13.9) | 0.467 |
| Aspirin, n (%) | | 45 (8.4) | 24 (9.0) | 21 (7.9) | 0.664 |
| **Renal Function Status** | |  |  |  |  |
| eGFR, median (IQR), ml/min/1.73 m^2^ ^a^ | | 9.4 (6.6-13.9) | 10.3 (6.8-14.0) | 9.2 (6.2-13.7) | 0.105 |
| Urine PCR, median (IQR), mg/g | | 1512 (1000-2475) | 1482 (989-2542) | 1512 (1010-2392) | 0.553 |
| **Laboratory Data** | |  |  |  |  |
| Hemoglobin, mean (SD), g/dL | | 9.2 (1.7) | 9.3 (1.8) | 9.1 (1.7) | 0.121 |
| Albumin, mean (SD), g/dL | | 3.9 (0.5) | 3.9 (0.5) | 3.9 (0.5) | 0.691 |
| Blood glucose, mean (SD), mg/dl | | 98.5 (16.2) | 97.4 (15.7) | 99.6 (16.6) | 0.037 |
| Total cholesterol, median (IQR), mg/dL | | 184 (153-213) | 185 (151-218) | 184 (153-209) | 0.550 |
| Triglyceride, median (IQR), mg/dL | | 112 (80-162) | 125 (82-171.5) | 103 (75-147) | 0.001 |
| C-reactive protein, median (IQR), mg/L | | 1.3 (0.5-5.7) | 1.2 (0.5-7.5) | 1.3 (0.5-4.3) | 0.996 |
| Hba1c, % | | 5.4 (0.6) | 5.4 (0.6) | 5.3 (0.6) | 0.224 |
| Sodium, mean (SD), mEq/L | | 138.0 (3.4) | 137.9 (3.8) | 138.2 (3.0) | 0.270 |
| Potassium, mean (SD), mEq/L | | 4.4 (0.7) | 4.5 (0.7) | 4.3 (0.6) | 0.061 |
| Phosphorus, mean (SD), mg/dL | | 5.1 (1.5) | 5.1 (1.5) | 5.1 (1.4) | 0.753 |
| Calcium, mean (SD), mg/dL | | 8.8 (0.8) | 8.9 (0.8) | 8.7 (0.9) | <0.001 |
| Bicarbonate, mean (SD), mEq/L | | 18.1 (4.1) | 18.5 (4.2) | 17.7 (4.0) | 0.073 |
| Uric acid, mean (SD), mg/dL | | 7.5 (2.0) | 7.9 (2.0) | 7.0 (1.7) | <0.001 |

Abbreviations: BMI, body mass index; CKD, chronic kidney disease; CRP, c-reactive protein; eGFR, estimated glomerular filtration rate; Hba1c, glycosylated hemoglobin; IQR, interquartile range; MBP, mean blood pressure; RAS, renin-angiotensin system; SD, standard deviation; UPCR: urine protein-creatinine ratio;

^a^ Obtained by using the Modification of Diet in Renal Disease study equation

*Supplementary Table 4.* Risk of clinical outcomes by glycosuria status in patients with non-diabetic stage 4-5 CKD after propensity score matching ^a^

|  |  | | |  | **Risk ^e^** | | | |
| --- | --- | --- | --- | --- | --- | --- | --- | --- |
|  |  | **Events (%)** | **Crude Event Rate (/100 patient-years) ^d^** |  | | **Unadjusted** | **Model 1** | **Fully adjusted model** |
| **ESRD** ^b^ |  |  |  |  | |  |  |  |
| Non-glycosuria |  | 151 (56.6) | 14.57 |  | | 1 (reference) | 1 (reference) | 1 (reference) |
| Glycosuria |  | 156 (58.4) | 13.08 |  | | 1.03 (0.82-1.28) | 0.75 (0.60-0.95) | 0.78 (0.62-0.98) |
| **Rapid Renal Function Decline ^c^** | |  |  |  | |  |  |  |
| Non-glycosuria |  | 70 (26.2) | 6.75 |  | | 1 (reference) | 1 (reference) | 1 (reference) |
| Glycosuria |  | 51 (19.1) | 4.28 |  | | 0.67 (0.45-1.01) | 0.63 (0.41-0.97) | 0.64 (0.41-0.99) |
| **Cardiovascular events** |  |  |  |  | |  |  |  |
| Non-glycosuria |  | 39 (14.6) | 3.76 |  | | 1 (reference) | 1 (reference) | 1 (reference) |
| Glycosuria |  | 35 (13.1) | 2.94 |  | | 0.95 (0.60-1.50) | 0.92 (0.58-1.46) | 0.88 (0.55-1.41) |
| **All-cause Mortality** |  |  |  |  | |  |  |  |
| Non-glycosuria |  | 46 (17.2) | 4.44 |  | | 1 (reference) | 1 (reference) | 1 (reference) |
| Glycosuria |  | 45 (16.9) | 3.77 |  | | 0.97 (0.64-1.46) | 1.03 (0.68-1.55) | 0.89 (0.58-1.36) |

Abbreviations: BMI, Body mass index; CKD, Chronic kidney disease; CI, Confidence interval; HR, Hazard ratio; UPCR, Urine protein-creatinine ratio.

^a^ Model 1 adjusts for age, gender, eGFR, log-transformed UPCR; fully adjusted model adjusts for covariates in model 1 plus cardiovascular disease history, mean blood pressure, hemoglobin, albumin, log-transformed CRP, BMI, log-transformed cholesterol, phosphorus, uric acid, potassium and bicarbonate.

^b^ ESRD includes long-term hemodialysis, peritoneal dialysis, and renal transplantation

^c^ defined as eGFR slope < -5 ml/min/1.73m^2^/year based on Kidney Disease Improving Global Outcomes (KDIGO) guideline

^d^ the median follow-up period is 3.1 years

^e^ Hazard ratio of ESRD, cardiovascular events, and all-cause mortality; odds ratio of rapid renal function decline
